# Aging Health and R&D for Healthy Longevity Must Be Included into the WHO Work Program

**DOI:** 10.14336/AD.2017.1120

**Published:** 2018-04-01

**Authors:** Ilia Stambler, Kunlin Jin, Stephanie Lederman, Nir Barzilai, S. Jay Olshansky, Emem Omokaro, Jane Barratt, Vladimir N. Anisimov, Suresh Rattan, Shaohua Yang, Michael Forster, Julie Byles

**Affiliations:** ^1^Department of Science, Technology and Society, Bar Ilan University, Israeli Longevity Alliance, and Vetek (Seniority) Association - the Senior Citizens Movement, Israel.; ^2^Department of Pharmacology and Neuroscience, Institute for Healthy Aging, University of North Texas, USA.; ^3^International Society on Aging and Disease, Texas, USA.; ^4^American Federation for Aging Research, New York, USA.; ^5^Institute for Aging Research, Albert Einstein College of Medicine, New York, USA.; ^6^The School of Public Health at the University of Illinois at Chicago, Illinois, USA.; ^7^African Society for Ageing Research and Development, and Gerontology Association of Nigeria, Nigeria; ^8^International Federation on Ageing, Toronto, Canada.; ^9^The Gerontological Society of the Russian Academy of Sciences and Russian Academy of Sciences, Saint-Petersburg, Russian Federation.; ^10^Biological Section, International Association of Gerontology and Geriatrics (European Region, IAGG-ER).; ^11^Department of Molecular Biology and Genetics, Aarhus University, Aarhus, Denmark.; ^12^International Longevity Centre-Australia, and Faculty of Health, The University of Newcastle, Australia

It can be confidently stated that global population aging is both the greatest success of global public health efforts of the past, as well as the greatest challenge for the further global public health efforts of the future.

Over the past decades, life expectancy at birth has increased globally, reaching a world-wide average of about 71.5 in 2017 (7 years longer than in 1990) and around 80 in the developed countries (compared to about 50 years in the developed countries in the early 20th century). This achievement occurred primarily due to advances in basic public health such as sanitation, clean water, waste disposal, temperature controlled living and working environments, and advances in medical technology -- all of which yielded large and rapid declines in communicable diseases. Beginning with the last quarter of the 20th century, progress made against chronic fatal diseases (especially heart disease) from new diagnostic tools, treatments, and improved behavioral risk factors (such as reductions in smoking), generated further increases in life expectancy, although at a slower pace. Currently, while the highest life expectancies are still found in the “developed” countries, the opportunity for rising longevity remains likely for “developing” world in the coming decades. Considering that both rising longevity and population aging are likely demographic events in the coming decades, by 2050 the proportion of people over 60 years is expected to double from about 13% to nearly 25%, which, in absolute terms, means an increase from 962 million to 2.1 billion people [1]. Rising longevity during the last 150 years is a testament to human ingenuity, and there is reason to believe further advances are possible.

Yet global population aging, and rising longevity are also accompanied by challenges and opportunities across all nations.

The number of patients suffering from chronic non-communicable age-related diseases are estimated at tens of millions, and are expected to strongly increase worldwide due to the rapid population aging. Thus, 36 million people worldwide are living with old-age dementia, and their numbers are expected to double every 20 years, reaching 66 million by 2030, and 115 million by 2050 [2]. According to World Health Organization’s data, “Noncommunicable diseases (NCDs) kill 40 million people each year, equivalent to 70% of all deaths globally. Cardiovascular diseases account for most NCD deaths, or 17.7 million people annually, followed by cancers (8.8 million), respiratory diseases (3.9 million), and diabetes (1.6 million).” Also, according to WHO data, “Each year, 15 million people die from a NCD between the ages of 30 and 69 years; over 80% of these "premature" deaths occur in low- and middle-income countries” [3]. In other words, of the 57 million deaths in the world each year, nearly 50% occur due to chronic non-communicable diseases in the world’s oldest population (70+), and over 60% in the older population (60+), making the health of older persons the worst and most urgent global health problem.

In view of the urgency of the problem, it seems highly surprising that in the forthcoming draft 13^th^ General Programme of Work of the World Health Organization for 2019-2023 - the issue of aging and aging-related ill health is excluded completely! Beside a cursory mention of the word “aging,” this work program does not contain any specific objectives, deliverables and actions to improve the health of the aged [4]. This means that, through 2023, according to this document, the World Health Organization is not obliged to provide any services to care for the health of older persons or to improve their health, not to mention conduct any research and development to create new therapies and technologies for improving the health of the aged. The issues of aged health are not in the WHO work program! This, in essence, appears to be a manifestation of “ageism” in health care and health research! The very cutoff at 70 years of age, discriminating “premature” from “mature” mortality, is questionable both scientifically and ethically. Does it imply that “mature” deaths should not be combated or reduced? Still, the complete exclusion of any actions for the improvement of aged health and reduction of aged mortality is not just unjustifiable, but inexplicable.

It should be obvious, not just to any health professional but to any lay person, that the global population aging poses grave and urgent challenges for global health, in the “developed” as well as the “developing” world. In the developing world, the faster population aging also means a faster increase in the incidence of chronic age-related NCD’s, while the geriatric and old-age NCD care and research capabilities in these countries may be under par and unprepared. Hence, in the “developing world” in particular, not addressing the problems of aging, not improving national geriatric care and research capabilities, will condemn millions of the world’s poorest and most disadvantaged older people to misery that could be avoided. This is one of the reasons the explicit exclusion of the health needs of the global aging population in the draft work program of the WHO appears to be inadmissible, even incredible.

Such an exclusion can have dire consequences for global health. First and foremost, it will signal to governments, in particular in developing countries, that aging health is not an important priority to be addressed with urgency. Practically, this attitude could result in many existing and future health care and health research programs on aging being eliminated. Even now, the issues related to aging health have not been strongly prioritized by WHO. Indicatively, as of 2016, the entire budget for the World Health Organization’s “Ageing and Health” program was $13.5M, out of about $4.4 billion total WHO budget (0.3%). No budget portion was specified for anything indicative of “ageing research” [5]. Now, with the explicit exclusion of aging health from the WHO work plan, will the “Ageing and Health” programs, and other ageing-related programs (both care and R&D) receive no funding and support whatsoever?

How can this exclusion coexist with the mission of WHO’s division on “Ageing and Life Course”? How can it coexist with the recently adopted WHO’s Global Strategy and Action Plan on Ageing and Health (GSAP) for 2016-2020, endorsed by all the WHO member states? According to its goal statement, the GSAP must prepare for the “Decade of Healthy Ageing from 2020 to 2030” which was also announced by WHO [6]. The coordination and consultation between various arms and branches of the WHO must improve. The developers of the WHO Work Program must avail of the world expertise on ageing health, within the WHO and externally, to develop an effective, strategically-minded and inclusive global health program.

Most importantly, we urge the WHO to include and emphasize specific tasks and goals to maintain and improve the health of the global aging population in its work program. Among others, these tasks and goals must include enhancing scientific research and technological development aimed to provide new effective and safe therapies to meet the pressing health needs of the global aging population [7].

We also urge the readers to make your voice heard, advocate and increase publicity about the need to include and implement concrete measures to improve aging health, including R&D for healthy longevity, as a priority in the WHO work program [8].
